# Continuous intraperitoneal insulin infusion in type 1 diabetes: a 6-year post-trial follow-up

**DOI:** 10.1186/1472-6823-14-30

**Published:** 2014-04-07

**Authors:** Peter R van Dijk, Susan JJ Logtenberg, Klaas H Groenier, Rijk OB Gans, Nanne Kleefstra, Henk JG Bilo

**Affiliations:** 1 Diabetes Centre, Isala, P.O. box 10400, 8000G.K Zwolle, The Netherlands; 2 Department of Internal Medicine, University of Groningen, University Medical Center Groningen, Groningen, The Netherlands; 3 Department of General Practice, University of Groningen, University Medical Center Groningen, Groningen, The Netherlands; 4 Langerhans Medical Research group, Zwolle, The Netherlands; 5 Isala, Department of Internal Medicine, Zwolle, The Netherlands

**Keywords:** Type 1 diabetes mellitus, Intraperitoneal insulin, Insulin infusion devices, Quality of life, Treatment satisfaction, Complications, Subcutaneous insulin

## Abstract

**Background:**

Continuous intraperitoneal insulin infusion (CIPII) with an implantable pump is a treatment option for patients with type 1 diabetes mellitus (T1DM). Aim of the present study was to describe the long-term course of glycaemic control, complications, health related quality of life (HRQOL) and treatment satisfaction among T1DM patients treated with CIPII.

**Methods:**

Nineteen patients that participated in a randomized cross-over trial comparing CIPII and subcutaneous (SC) therapy in 2006 were followed until 2012. Laboratory, continuous glucose monitoring, HRQOL and treatment satisfaction measurements were performed at the start of the study, the end of the SC-, the end of the CIPII treatment phase in 2006 and during CIPII therapy in 2012. Linear mixed models were used to calculate estimated values and to test differences between the moments in time.

**Results:**

In 2012, more time was spent in hyperglycaemia than after the CIPII treatment phase in 2006: 37% (95% CI 29, 44) vs. 55% (95% CI 48, 63), mean difference 19.8% (95% CI 3.0, 36.6). HbA1c was 65 mmol/mol (95% CI 60, 71) at the end of the SC treatment phase in 2006, 58 mmol/mol (95% CI 53, 64) at the end of the CIPII treatment phase and 65 mmol/mol (95% CI 60, 71) in 2012, respectively (p > 0.05). In 2012, the median number of grade 2 hypoglycaemic events per week (1 (95% CI 0, 2)) was still significantly lower than during prior SC therapy (3 (95% CI 2, 4)): mean change -1.8 (95% CI -3.4, -0.4). Treatment satisfaction with CIPII was better than with SC insulin therapy and HRQOL remained stable. Pump or catheter dysfunction of the necessitated re-operation in 7 patients. No mortality was reported.

**Conclusions:**

After 6 years of CIPII treatment, glycaemic regulation is stable and the number of hypoglycaemic events decreased compared to SC insulin therapy. Treatment satisfaction with CIPII is superior to SC insulin therapy, HRQOL is stable and complications are scarce. CIPII is a safe and effective treatment option for selected patients with T1DM, also on longer term.

## Background

The mainstay of type 1 diabetes mellitus (T1DM) treatment consists of subcutaneous (SC) insulin administration using multiple daily injections (MDI) or continuous subcutaneous insulin infusion (CSII) with an externally placed pump. Although most patients achieve acceptable glycaemic control using MDI or CSII, a relatively small group of patients fails to reach adequate glycaemic control, have frequent hypoglycaemic episodes or SC insulin resistance, despite intensive SC insulin therapy. For these patients, continuous intraperitoneal insulin infusion (CIPII) with an implantable pump is a treatment option [1].

With intraperitoneal administration, insulin is better absorbed and allows blood glucose levels to return to baseline values more rapidly with more predictable insulin profiles compared to SC insulin administration [2,3]. The higher hepatic uptake of insulin mitigates peripheral plasma insulin concentrations compared to SC administration [3,4]. Other possible effects include improvement of the impaired glucagon and hepatic glucose production in response to hypoglycaemia through alleviation of peripheral hyperinsulinaemia [5].

In 2006, a randomized, cross-over study was performed at our centre to investigate the effects of CIPII on the risk of hypoglycaemia, compared to intensive SC insulin treatment, both for a six-month period. Glycaemic control, health related quality of life (HRQOL) and treatment satisfaction improved during CIPII treatment as compared to SC insulin administration and there was no reduction or increase in hypoglycaemic events [6,7]. After the study all participants chose to continue CIPII.

Aim of the current analysis is to investigate long-term glycaemic control, HRQOL, treatment satisfaction and complications among patients with T1DM, treated with CIPII.

## Methods

### Study population

Twenty three patients with T1DM, low fasting C-peptide concentrations (<0.20 nmol/l) and intermediate or poor glycaemic control, defined as HbA1c ≥58 mmol/mol and/or ≥5 incidents of hypoglycaemia (<4.0 mmol/l) per week, who were aged 18–70 years and treated with SC insulin, were included in the cross-over study in 2006. The exclusion criteria were: impaired renal function (plasma creatinine ≥150 μmol/L or glomerular filtration rate ≤50 ml/min), cardiac problems (unstable angina or myocardial infarction within the previous 12 months or New York Heart Association class III or IV congestive heart failure), mentally handicapped, current or past psychiatric treatment for schizophrenia, cognitive or bipolar disorder, current use or oral corticosteroids or suffering from a condition which necessitated oral or systemic corticosteroids use more than once in the previous 12 months, substance abuse, other than nicotine, current pregnancy or plans to become pregnant during the trial, plans to engage in activities that require going >25 feet below sea level. After the cross-over study all patients chose to continue CIPII with an implantable pump (Minimed Insulin Pump).

### Study design

The previous study (NCT00286962) started in 2006, had an open-label, randomized cross-over design and was performed at Isala (Zwolle, the Netherlands). The study consisted of 4 phases: the qualification phase, the first treatment phase, the crossover phase, and the second treatment phase. After a 3-month qualification phase, patients were randomly allocated to one of two groups, which differed only in the sequence of the two therapies. Between both treatment phases of 6 months, a crossover phase of 4 weeks was instituted to minimize the carryover effects of CIPII. The results of this study were reported previously and showed a significant decrease in HbA1c, with more time spent in euglycaemia and without a change in hypoglycaemic events with CIPII as compared to SC insulin therapy. In addition, HRQOL and treatment satisfaction improved with CIPII. [6,7]. Follow-up measurements for the present analysis were performed in December 2012 until March 2013.

### Procedures and methods

At the start of the 2006 cross-over study, 3 patients were on MDI and 20 on CSII. During the SC treatment phase in the 2006 study, SC insulin was delivered with either MDI or CSII, according to what was used prior to the study. Patients treated with MDI continued to use their own insulin regime, i.e. rapid acting insulin analogues before meals and a daily dose of long acting insulin. Patients treated with CSII used rapid acting insulin analogues. During the crossover phase insulin was administered SC. If the subject was using more than 40 IU of SC insulin per day prior to starting the CIPII phase of the study, his or her starting dose was set at 90% of the prior SC dose. Subjects using less than 40 IU of SC insulin received a starting dose of 80% of the prior SC dose. Initially the dose was equally divided between a basal rate (50%) and a bolus before meals [8].

**Figure 1:**
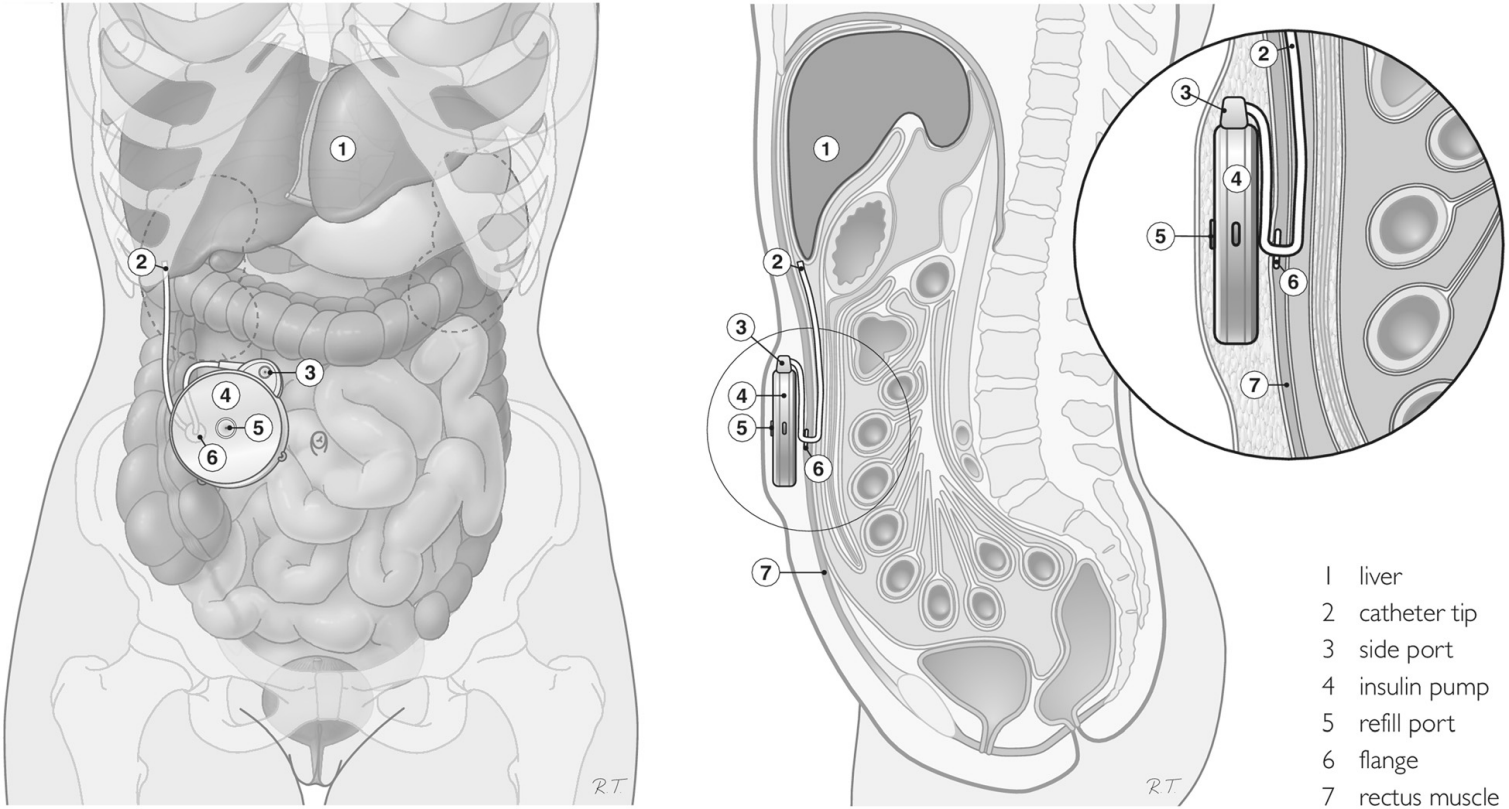
**Legend: Illustration of the implantable pump in situ (derived from **[9]**).**

In 2006–2007, the CIPII pump was implanted under general anaesthesia at the start of the CIPII phase in all subjects. Insulin (U-400 HOE 21PH, semi synthetic human insulin of porcine origin, trade name: Insuplant® Hoechst, Frankfurt, Germany, nowadays Sanofi-Aventis) was administered with the implantable pump. Since there were no batches left of the U400 semi synthetic human insulin, a new human recombinant insulin (400 IU/ml; human insulin of E. Coli origin, trade name: Insuman Implantable®, Sanofi-Aventis) was used from 2010 onwards. Between 2006 and 2012, all patients received standard care at our outpatient clinic which consisted of insulin refills every 6–12 weeks and an rinse procedure with NaOH was performed every 9 months or in case of insulin underdelivery. The insulin pump, implantation, insulin dosage and refill procedures have been described in more detail previously [9,8]. An illustration of the implantable pump is provided in Figure 1.

### Measurements

In order to yield information about the long-term impact of CIPII on glycaemic control in comparison to that on SC insulin therapy, we compared data derived from the measurements in 2012/2013 with data from the start of the 2006 study, the end of the SC-, the end of the CIPII phase of the 2006 cross-over study.

Demographic and clinical parameters included smoking and alcohol habits, year of diagnosis of diabetes, presence of complications, any comorbidity, height and weight, daily insulin dose, number of self-reported hypoglycaemic events grade 1 (<4.0 mmol/L) and grade 2 (<3.5 mmol/L) during the last 7 days. The HbA1c level was measured with a Primus Ultra2 system using high-performance liquid chromatography (reference value 20–42 mmol/mol). In addition, 5- to 7-day 24-hours interstitial glucose profiles were recorded with a continuous glucose monitoring (CGM) system (iPro2, Medtronic, Northridge, CA, USA). Time spent in the hypoglycaemic range was defined as the percentage of CGM recordings <4.0 mmol/L, time spent in euglycaemic range was defined as the percentage of CGM recordings from 4.0 to 10.0 mmol/L, and time spent in hyperglycaemic range was defined as the percentage of CGM recordings >10.0 mmol/L.

For HRQOL assessment, the 36-item short-form health survey (SF-36) and the World Health Organization-Five Well-Being Index (WHO-5) questionnaires were used. The SF-36 is a widely used, generic questionnaire with 36 items involving eight subscales and a physical and mental component summary (PCS and MCS, respectively). Scale scores range from 0–100, with higher scores indicating better HRQOL [10,11]. The WHO-5 is designed to measure positive well-being and is reported to be better in identifying depression than the MCS [12,13]. It consists of five items with a total score ranging from 0–100. A total score below 50 or answer of ’0 or 1’ suggests poor emotional well-being [14]. Treatment satisfaction was measured with the Diabetes Treatment Satisfaction Questionnaire (DTSQs). All eight items are scored on a 7-point scale. Two items assess perceived frequency of hyperglycaemia and hypoglycaemia, and six items comprise the treatment satisfaction scale, with higher scores indicating higher satisfaction (range 0–36) [15].

### Statistical analysis

Descriptive summaries included the mean with standard deviation (SD) for normally distributed variables and the median with the interquartile range (25th-75th percentile) for other variables. Q-Q plots were used to determine if the tested variable had a normal distribution or not. Time variables, such as times spent in the different glycaemic states, are presented as absolute values. Linear mixed models with Bonferroni correction were used to calculate and to test differences in time. Estimated values and estimated differences, calculated with linear mixed models, are reported. All observed values are presented in Additional file 1. All statistical analysis were performed with SPSS software (version 20.0, Inc, Chicago, Il, USA). A two-sided significance level of 0.05 was considered statistically significant.

### Ethical considerations

Studies were performed in accordance with the Declaration of Helsinki. For this study, informed consent was obtained from all patients in 2006 as well as in 2012. Approval by the medical ethics committee of the Isala (Zwolle, The Netherlands) was given for the crossover study in 2006 and the follow-up measurements in 2012.

## Results

### Patients

Of 23 patients who participated in the previous cross-over study, 22 were still treated with CIPII in 2012. One patient stopped CIPII treatment due to neuropathic pains. The patient believed the implanted pump caused this pain. Two female patients were excluded from the current analysis: 1 due to chronic prednisolone use for myasthenia gravis and 1 due to participation in an in vitro fertilization program. One patient refused participation. Therefore, 19 patients (53% male) are included in the present analysis, with a mean age of 45 (10) years and a diabetes duration 23 (16, 33) years at the start of the 2006 study. Four of these patients are current smokers.

### Clinical parameters

The estimated values of the clinical parameters and comparisons between the start of the 2006 study, the end of the SC-, the end of the CIPII treatment phase and the start of the present 2012 study, 6 (0.4) years later, are presented in Table 1. Systolic blood pressure, BMI, cholesterol and the insulin dose remained stable over time. Two patients were diagnosed with neuropathy, one with retinopathy and one with a macrovascular complication (occlusion of the femoral artery). There were no new cases of nephropathy.

**Table 1:** Clinical and glycaemic parameters

	**Start 2006 study (A)**	**End SC phase (B)**	**End CIPII phase (C)**	**2012 study (D)**	**D vs. A**	**D vs. B**	**D vs. C**
**Clinical parameters**							
SBP (mmHg)	141 (133, 150)	135 (126, 143)	139 (130, 147)	140 (131, 149)	-1. -1.1 (-17.6, 15.5)	5.5 (-11.0, 22.0)	1.3 (-15.2, 17.9)
BMI (kg/m^2^)	26 (24, 29)	27 (24, 29)	28 (25, 30)	26 (24, 29)	-0.4 (-4.9, 4.1)	-0.2 (-5.3, 4.9)	-1.2 (-6.1, 3.7)
Total cholesterol	4.8 (4.5, 5.2)	4.7 (4.3, 5.1)	4.5 (4.1, 4.9)	4.8 (4.4, 5.2)	-0.1 (-0.9, 0.7)	0.0 (-0.7, 0.8)	-0.2 (-0.5, 1.0)
HDL cholesterol	1.8 (1.6, 2.0)	1.8 (1.5, 2.0)	1.6 (1.3, 1.8)	1.7 (1.4, 1.9)	-0.1 (-0.6, 0.3)	-0.1 (-0.6, 0.4)	0.1 (-0.3, 0.6)
LDL cholesterol	2.6 (2.3, 3.0)	2.5 (2.2, 2.8)	2.4 (2.1, 2.7)	2.8 (2.5, 3.1)	0.2 (-0.4, 0.8)	0.3 (-0.3, 0.9)	0.4 (-0.2, 1.1)
Triglycerides	0.9 (0.7, 1.2)	1.1 (0.8, 1.3)	1.3 (1.0, 1.5)	1.0 (0.7, 1.3)	-0.1 (-0.5, 0.6)	-0.1 (-0.6, 0.5)	-0.3 (-0.8, 0.3)
Total insulin dose (IU/day)	19 (13, 24)	25 (20, 31)	20 (15, 25)	20 (15, 26)	8.2 (-18.3, 34.7)	8.2 (-17.6, 33.9)	6.1 (-19.7, 31.8)
Basal insulin dose (IU/day)	35 (24, 45)	32 (22, 43)	35 (25, 46)	44 (34, 55)	9.6 (-10.6, 29.8)	12.0 (-8.1, 32.2)	8.5 (-11.7, 28.7)
Bolus insulin dose (IU/day)	54 (40, 67)	58 (44, 71)	56 (42, 69)	65 (51, 78)	1.4 (-8.9, 11.7)	-5.1 (-15.3, 5.2)	0.4 (-9.9, 10.7)
**Glycaemic parameters**							
HbA1c (mmol/mol)	70 (64, 75)	65 (60, 71)	58 (53, 64)	65 (60, 71)	-4.5 (-14.9, 5.9)	-0.1 (-10.5,10.3)	7.1 (-3.3, 17.5)
Hypoglycaemia grade 1 †	4 (3, 6)	4 (3, 6)	4 (2, 5)	3 (1, 4)	-1.8 (-4.2, 0.7)	-1.7 (-4.2, 0.8)	-1.1 (-3.6, 1.4)
Hypoglycaemia grade 2 ‡	3 (2, 4)	3 (2, 4)	2 (2, 3)	1 (0, 2)	-1.8 (-3.4,-0.4)*	-1.9 (-3.5, -0.4)*	-1.4 (-3.0, 0.1)
Time spent in hypoglycaemia (%)	8 (5, 11)	8 (5, 11)	6 (3, 9)	5 (2, 7)	-3.7 (-9.3, 1.9)	-3.6 (-9.2, 2.0)	-1.1 (-6.7, 4.5)
Time spent in hyperglycaemia (%)	45 (36, 54)	47 (38, 56)	39 (30, 48)	59 (50, 68)	13.7 (-3.1, 30.5)	12.0 (-4.8, 28.8)	19.8 (3.0, 36.6)*
Time spent in euglycaemia (%)	47 (39, 54)	45 (38, 53)	55 (48, 63)	37 (29, 44)	-10.0 (-24.6, 4.6)	-8.4 (-23.0, 6.2)	-18.7 (-33.3, -4.1)*

Estimated values and differences are reported. Data are presented as estimated mean (95% CI) or mean change (95% CI). Numbers may not add up due to rounding. BMI; Body Mass Index, CIPII; continuous intraperitoneal insulin infusion, SBP; systolic blood pressure, SC; subcutaneous. *p < 0.05. † Defined as the number of blood glucose values <4.0 mmol/L per week. ‡ Defined as the number of blood glucose values <3.5 mmol/L per week.

### Glycaemic parameters

As shown in Table 1, the mean estimated HbA1c in 2012 was 65 (95% confidence interval (CI) 60, 71) mmol/mol and was not significantly different from the HbA1c at the start of the 2006 study: 70 (95% CI 64, 75) mmol/mol, with a mean estimated change of -4.5 mmol/mol (95% CI -14.9, 5.9; p = 1.0). Although there was a tendency to rise, the HbA1c in 2012 did not differ significantly from the HbA1c at the end of the SC phase (-0.1 mmol/mol 95% CI -10.5, 10.3; p = 1.0) and the end of the CIPII phase (7.1 mmol/mol 95% CI -3.3, 17.5; p = 0.4) of the 2006 study.

The number of grade 2 hypoglycaemic events per week decreased from 3 (95% CI 2, 4), at the start and at the end of the SC therapy phase of the 2006 study, to 1 (95% CI 0, 2) event per week in 2012. In 2012, compared with the start of the 2006 study the mean change was -1.8 events per week (95% CI -3.4,-0.4; p = 0.008) and compared with the end of SC therapy phase the mean change was -1.9 (95% CI -3.5, -0.4; p = 0.007). More time was spent in hyperglycaemia during CGM measurements in 2012 than at the end of the CIPII phase in 2006: mean change 19.8 (95% CI 3.0, 36.6; p = 0.013). Percentage time spent in euglycaemia with CIPII in 2012 was less than at the end of the CIPII phase of the 2006 study: mean change -18.7% (95% CI -33.3, -4.1; p = 0.005).

### HRQOL and Treatment satisfaction

As shown in Table 2, none of the SF-36 subscales and summary scores changed over time. The WHO-5 scores in 2012 remained stable over the years with CIPII. In 2012, 8 patients had a poor emotional well-being according to the WHO-5, compared to 9 at the end of the SC phase and 2 at the end of the CIPII study phase. The treatment satisfaction remained significantly higher with CIPII than with SC insulin: the mean difference between 2012 and the start of the 2006 study was 8.3 (95% CI 2.3, 14.3; p = 0.001) and between 2012 and the end of the SC phase was 8.4 (95% CI 2.4, 14.3; p = 0.001). The perceived hyperglycaemia score of the DTSQ was higher in 2012 than at the end of the 2006 CIPII therapy phase: 1.5 (95% CI 0.2, 2.7; p = 0.01).

**Table 2:** HRQOL and treatment satisfaction

	**Start 2006 study (A)**	**End SC phase (B)**	**End CIPII phase (C)**	**2012 study (D)**	**D vs. A**	**D vs. B**	**D vs. C**
**SF-36 subscales**							
Physical functioning	76 (66, 86)	69 (59, 79)	81 (71, 91)	76 (65, 86)	-0.3 (-19.7, 19.1)	7.4 (-12.1, 26.8)	-5.3 (-24.7, 14.1)
Social functioning	68 (53, 76)	65 (53, 76)	77 (66, 88)	74 (63, 85)	6.6 (-14.7, 27.8)	9.9 (-11.4, 31.2)	-2.6 (-32.9, 18.6)
Role limitations-physical	38 (17, 59)	42 (21, 63)	66 (45, 87)	57 (36, 78)	18.4 (-22.0, 58.8)	14.5 (-26.0, 54.9)	-9.2 (-49.6, 31.2)
Role limitations-emotional	68 (50, 87)	68 (50, 87)	86 (67, 100)	77 (58, 96)	8.8 (-27.3, 44.8)	8.8 (-27.3, 44.8)	-8.8 (-44.8, 27.3)
Mental health	70 (60, 79)	67 (58, 77)	77 (68, 87)	79 (70, 89)	9.6 (-8.1, 27.4)	12.0 (-5.8, 29.7)	2.1 (-15.7, 19.8)
Vitality	48 (39, 58)	43 (34, 71)	62 (52, 71)	58 (49, 67)	9.5 (-7.9, 26.9)	15.3 (-2.2, 32.7)	-4.5 (-32.9, 12.8)
Bodily pain	64 (52, 75)	64 (53, 76)	66 (54, 77)	67 (56, 78)	3.3 (-18.5, 25.0)	2.6 (-19.2, 24.3)	1.4 (-20.4, 23.1)
General health	41 (32, 50)	46 (37, 54)	56 (47, 64)	48 (38, 56)	6.5 (-10.1, 23.1)	1.7 (-14.9, 18.3)	-8.0 (-24.7, 8.6)
**SF-36 summary scores**							
Physical component score	56 (46, 65)	55 (45, 64)	68 (58, 77)	63 (54, 73)	7.3 (-10.9, 25.6)	8.5 (-9.7, 26.8)	-4.7 (-23.0, 13.5)
Mental component score	59 (50, 68)	58 (49, 67)	72 (63, 81)	67 (58, 76)	8.2 (-9.1, 25.5)	9.5 (-7.7, 26.8)	-4.4 (-21.6, 12.9)
**WHO-5 score**	49 (39, 59)	47 (37, 57)	69 (59, 79)	60 (50, 70)	10.5 (-9.0, 30.0)	12.6 (-6.9, 32.1)	-9.2 (-28.8, 10.2)
**DTSQ**							
Total score	24 (21, 27)	24 (21, 27)	33 (30, 36)	33 (29, 36)	8.3 (2.3, 14.3)*	8.4 (2.4,14.3)*	-0.3 (-6.3, 5.7)
Perceived hypoglycaemia score	5 (4, 6)	5 (4, 5)	2 (2, 3)	4 (3, 5)	-0.4 (-2.0, 1.2)	-0.8 (-2.4, 0.8)	0.3 (-1.3, 1.9)
Perceived hyperglycaemia score	3 (2, 4)	4 (3, 5)	3 (2, 3)	3 (2, 4)	-1.1 (-2.4, 0.1)	-0.9 (-2.1, 0.3)	1.5 (0.2, 2.7)*

Estimated values and differences are reported. Data are presented as estimated mean (95% CI) or mean change (95% CI). Numbers may not add up due to rounding. DTSQ; Diabetes Treatment Satisfaction Questionnaire, CIPII; continuous intraperitoneal insulin infusion, SC; subcutaneous, SF-36; 36-item short-form health survey, WHO-5; World Health Organization-Five Well-Being Index *p < 0.05.

### Device complications

After a mean duration of 5 (1.0) years, 3 cases of pump dysfunction and 3 cases of (expected) battery end-of-life necessitated replacement of the pump. In 3 patients a laparoscopic procedure was performed to replace the catheter and in 1 patient a laparoscopic operation was necessary to remove a fibrin plug from the tip of the catheter. The mean duration of hospital admission for the 10 patients who experienced any pump related issue (including planned replacement due to battery end-of-life) was 0.6 (0, 1) days per year. No mortality was reported.

## Discussion

After 6 years of treatment with CIPII, HbA1c leveled with the value these T1DM patients had during intensive SC therapy, prior to starting CIPII. Nevertheless, patients experienced significant less grade 2 hypoglycaemic events and remained much more satisfied with CIPII compared to the SC treatment.

During the previous cross-over trial in which CIPII was commenced there was a significant decrease in HbA1c compared to the SC treatment phase from 70 to 58 mmol/mol. Compared to the SC treatment phase, the decrease in that study was significantly greater with CIPII with a mean difference of 8.4 mmol/mol. During the follow-up period described in the present study HbA1c stabilized at a level of 65 mmol/mol, which was not different to the levels prior and shortly after starting CIPII 6 years before. Several studies have described the effect of CIPII, as compared to SC insulin therapy, on glycaemic control. In all 3 short-term randomized studies, HbA1c improved with CIPII [6,16,17]. In contrast to the findings in the present study, HbA1c improvement persisted over the years in subsequent long-term observational studies. Nevertheless, follow-up duration (45 days to 7.3 years) varied substantially between studies and, importantly, not all patients in those studies had intermediately or poorly controlled T1DM (HbA1c 63 to 83 mmol/mol) [18-24].

In line with previous studies, the number of grade 2 hypoglycaemic events decreased during CIPII in the present cohort as compared to prior SC therapy [16,20,25]. This may well be the result of a slightly more hyperglycaemic profile. Although speculative, the restoration of the portal to peripheral insulin gradient with CIPII treatment, known to improve glucagon secretion and hepatic glucose production in response to hypoglycaemia, may also help to explain this finding [5,26].

The HbA1c course in the current cohort may be partly explained by the effect of being under strict study conditions during the cross-over study, which diminishes after the end of the study. Several other explanations may be taken into account. First, complications of CIPII may also have a negative influence on glycaemic regulation. Second, it should be mentioned that from 2010 onwards all CIPII patients switched to another insulin (Insuman® Implantable 400 IU/mL) because the previous insulin batch (U-400 HOE 21PH, Insuplant® 400 IU/mL) was no longer available. The effect of the change in insulin formulation remains to be determined from an on-going study (clinical trials identifier NCT01194882).

The switch from SC insulin to CIPII increases HRQOL, which stabilizes over time [7,20]. In the present study the level of HRQOL among CIPII treated subjects perpetuated. Nevertheless, as found in other studies and underlined by the fact that 42% of all patients had a WHO-5 score indicating poor emotional well-being, the HRQOL of these individuals remains poor [1,19,20]. We found the SF-36 subscales role-physical and vitality to be comparable to patients with a minor (uncomplicated) chronic disease and the other subscales similar to patients with complicated diabetes or complicated coronary artery disease [27]. Still, it is likely that the short duration of hospital admissions found in the present study, compared to 45 days per year before implantation of the pump previously described in a similar population, positively influence HRQOL and treatment satisfaction [19].

Since CIPII is used as a last treatment option in the Netherlands, the population in the present study is complex, strictly selected, and has a small size. On the other hand this limitation reflects general practice nowadays where CIPII is limited to a small number of patients in a small number of centers. Furthermore, when interpreting the comparisons between CIPII and previous SC therapy made in this study one should take differences in treatment periods (e.g. a duration of 6 months of the SC phase during a controlled study versus 6.4 years of subsequent CIPII therapy) into account. Prospective, long-term and large-scale studies with respect to i.e. glycaemic control, HRQOL and cost-effectiveness to compare SC and CIPII therapy for T1DM are imperative.

## Conclusions

Taken together, the stable HRQOL, increased treatment satisfaction, little time spent in hospital and stable HbA1c combined with a decrease in grade 2 hypoglycaemic events as compared to previous SC therapy underlines the clinical observation that CIPII is a valuable treatment option for selected patients with T1DM, also on longer term.

## Abbreviations

CGM: Continuous glucose monitoring; CIPII: Continuous intraperitoneal insulin infusion; CSII: Continuous subcutaneous insulin infusion; DTSQ: Diabetes Treatment Satisfaction Questionnaire; HRQOL: Health related quality of life; MCS: Mental component summary; MDI: Multiple daily injections; PCS: Physical component summary; SC: Subcutaneous; SF-36: 36-item short-form health survey; WHO-5: World Health Organization-Five Well-Being Index.

## Competing interests

Disclosure summary: The authors have nothing to disclose.

Sponsoring: The iPro2 glucose monitoring systems and Enlite sensors for the continuous glucose measurements were sponsored by Medtronic International Trading Sarl (Switzerland). Medtronic had no influence whatsoever in the design of the study, data collection, data analysis and interpretation of the data.

## Authors’ contributions

PD: researched data and wrote the manuscript. SL researched data and contributed to writing the manuscript. KG: researched data. RG NK and HB all contributed to writing the manuscript. All authors read and approved the final manuscript.

## Pre-publication history

The pre-publication history for this paper can be accessed here:

http://www.biomedcentral.com/1472-6823/14/30/prepub

## Supplementary Material

Additional file 1 Observed values at the different moments in time. Click here for file
