# Supplementary material for: Continuous intraperitoneal insulin infusion in type 1 diabetes: a 6-year post-trial follow-up
Source: BMC Endocr Disord. 2014 Apr 7;14:30. doi: 10.1186/1472-6823-14-30 (PMC4029992; doi:10.1186/1472-6823-14-30)
Supplement: Additional file 1 — Observed values at the different moments in time. [file 1472-6823-14-30-S1.docx]

**Additional file 1.** Observed values at the different moments in time

|  | **Start 2006 study** | **End SC phase** | **End CIPII phase** | **2012 study** |
| --- | --- | --- | --- | --- |
| **Clinical parameters** |  |  |  |  |
| SBP (mmHg) | 141 (21) | 135 (18) | 139 (19) | 140 (17) |
| BMI (kg/m^2^) | 26.6 (5.2) | 26.5 (4.8) | 27.5 (5.2) | 27.5 (4.5) |
| Total cholesterol | 4.8 (0.8) | 4.7 (0.9) | 4.5 (0.9) | 4.8 (1.0) |
| HDL cholesterol | 1.8 (0.5) | 1.7 (0.5) | 1.6 (0.5) | 1.7 (0.6) |
| LDL cholesterol | 2.7 (0.7) | 2.5 (0.7) | 2.4 (0.6) | 2.8 (0.8) |
| Triglycerides | 0.9 (0.4) | 1.1 (0.6) | 1.2 (0.8) | 1.0 (0.5) |
| Total insulin dose (IU/day) | 50 (35, 75) | 50 (40, 68) | 49 (35, 70) | 57 (46, 74) |
| Basal insulin dose (IU/day) | 28 (22, 31) | 26 (13,37) | 30 (20, 52) | 37 (26, 60) |
| Bolus insulin dose (IU/day) | 16 (10, 25) | 21 (13,37) | 16 (13, 30) | 17 (13, 28) |
| **Glycaemic parameters** |  |  |  |  |
| HbA1c (mmol/mol) | 70 (12) | 65 (13) | 58 (9) | 65 (13) |
| Hypoglycaemia grade 1 † | 4 (2, 5) | 4 (1, 7) | 3 (2, 5) | 2 (0, 3) |
| Hypoglycaemia grade 2 ‡ | 3 (1, 4) | 3 (1, 4) | 2 (1, 3) | 1 (0, 2) |
| Time in hypoglycaemia (%) | 8 (7) | 8 (8) | 6 (6) | 5 (5) |
| Time in hyperglycaemia (%) | 45 (16) | 47 (20) | 39 (19) | 59 (20) |
| Time in euglycaemia (%) | 47 (12) | 45 (16) | 55 (18) | 36 (19) |
| **SF-36** |  |  |  |  |
| Physical functioning | 76 (20) | 69 (24) | 81 (21) | 76 (23) |
| Social functioning | 68 (21) | 65 (29) | 77 (25) | 74 (21) |
| Role limitations-physical | 38 (11) | 42 (11) | 66 (11) | 57 (11) |
| Role limitations-emotional | 68 (10) | 68 (9) | 86 (9) | 77 (9) |
| Mental health | 70 (24) | 67 (22) | 77 (17) | 79 (17) |
| Vitality | 48 (22) | 43 (21) | 62 (19) | 58 (18) |
| Bodily pain | 64 (25) | 64 (29) | 66 (23) | 67 (21) |
| General health | 41 (18) | 46 (21) | 56 (19) | 48 (17) |
| Physical component score | 56 (18) | 55 (24) | 69 (20) | 63 (21) |
| Mental component score | 59 (20) | 58 (22) | 72 (19) | 67 (17) |
| **WHO-5-score** | 50 (21) | 48 (25) | 70 (21) | 60 (22) |
| **DSTQ** |  |  |  |  |
| Total score | 24 (8) | 23 (9) | 33 (4) | 32 (3) |
| Perceived hypoglycaemia score | 3 (2) | 4 (2) | 3 (2) | 3 (1) |
| Perceived hyperglycaemia score | 5 (1) | 5 (1) | 2 (2) | 4 (2) |

Data are presented as mean (SD), median (25^th^-75^th^ percentile). Observed values are reported. BMI; Body Mass Index, CIPII; continuous intraperitoneal insulin infusion, SBP; systolic blood pressure, SC; subcutaneous. *p<0.05. † Defined as a number of blood glucose value <4.0 mmol/L per week. ‡ Defined as a number of blood glucose value <3.5 mmol/L per week.
